# Flaviviral Replication Complex: Coordination between RNA Synthesis and 5′-RNA Capping

**DOI:** 10.3390/v7082837

**Published:** 2015-08-13

**Authors:** Valerie J. Klema, Radhakrishnan Padmanabhan, Kyung H. Choi

**Affiliations:** 1 Department of Biochemistry and Molecular Biology, Sealy Center for Structural Biology and Molecular Biophysics, University of Texas Medical Branch at Galveston, Galveston, TX 77555-0647, USA; E-Mail: vaklema@utmb.edu; 2 Department of Microbiology and Immunology, Georgetown University School of Medicine, Washington, DC 20057, USA; E-Mail: rp55@georgetown.edu

**Keywords:** viral replication complex, flavivirus, RNA-dependent RNA polymerase, RNA synthesis, 5′-RNA capping, NS5, NS3

## Abstract

Genome replication in flavivirus requires (−) strand RNA synthesis, (+) strand RNA synthesis, and 5′-RNA capping and methylation. To carry out viral genome replication, flavivirus assembles a replication complex, consisting of both viral and host proteins, on the cytoplasmic side of the endoplasmic reticulum (ER) membrane. Two major components of the replication complex are the viral non-structural (NS) proteins NS3 and NS5. Together they possess all the enzymatic activities required for genome replication, yet how these activities are coordinated during genome replication is not clear. We provide an overview of the flaviviral genome replication process, the membrane-bound replication complex, and recent crystal structures of full-length NS5. We propose a model of how NS3 and NS5 coordinate their activities in the individual steps of (−) RNA synthesis, (+) RNA synthesis, and 5′-RNA capping and methylation.

## 1. Flavivirus Genome and Viral Non-Structural (NS) Proteins

Flaviviruses are positive (+) sense RNA viruses belonging to the family *Flaviviridae*, which also includes *Hepacivirus* and *Pestivirus*. The *Flavivirus* genus includes over 70 viruses, many of which cause arboviral diseases in humans, such as dengue (DENV), West Nile (WNV), tick-borne encephalitis (TBEV), and yellow fever virus. The 11 kb flaviviral RNA genome consists of a 5′-cap, a 5′-untranslated region (5′-UTR), a single open reading frame (ORF), and a 3′-UTR. The 5′- and 3′-UTRs contain conserved RNA secondary structures that are important for viral replication, including sequences that mediate long range 5′- and 3′-RNA interactions [1,2,3,4,5,6,7,8]. The viral ORF is translated into a polyprotein, C-prM-E-NS1-NS2A-NS2B-NS3-NS4A-NS4B-NS5, that is subsequently cleaved into individual proteins by viral and host proteases. Three structural proteins (C, prM, and E) form capsids and seven non-structural (NS) proteins (NS1, NS2A, NS2B, NS3, NS4A, NS4B, NS5) are involved in the assembly of the viral replication complex [9]. Among NS proteins, the functions of NS3 and NS5 in viral replication are well established. NS3 consists of an N-terminal serine protease and a C-terminal helicase. NS3 protease activity requires NS2B as a cofactor, and cleaves the viral polyprotein at several positions between NS proteins [10,11,12,13,14]. The NS3 helicase domain has helicase, RNA-stimulated nucleoside triphosphate hydrolase and 5′-RNA triphosphatase activities [15,16,17,18,19]. Helicase activity would be required for unwinding the double-stranded (ds) RNA intermediate formed during genome synthesis, and 5′-RNA triphosphatase activity is required for 5′-RNA cap formation [20,21]. NS5, the largest viral protein (103 kDa), consists of an N-terminal methyltransferase (MTase) and a C-terminal RNA-dependent RNA polymerase (RdRp). The RdRp is involved in viral genome replication and carries out both (−) and (+) strand RNA synthesis [22]. The NS5 MTase has RNA guanylyltransferase and methyltransferase activities necessary for 5′-RNA capping and cap methylations [23,24]. Little is known about functions of the membrane proteins NS2A, NS4A, and NS4B, but they are likely involved in membrane alterations and assembly of the viral replication complex on the cellular membrane [25,26,27,28,29]. NS1 may be involved in multiple steps in the viral life cycle, including viral replication [30,31,32,33]. NS1 exists as two forms, either a membrane-bound dimer in the viral replication complex or a secreted hexameric form with as-yet-unknown function [34,35]. More extensive reviews of NS protein functions can be found elsewhere [36,37].

## 2. Flavivirus Genome Replication

**Figure 1:**
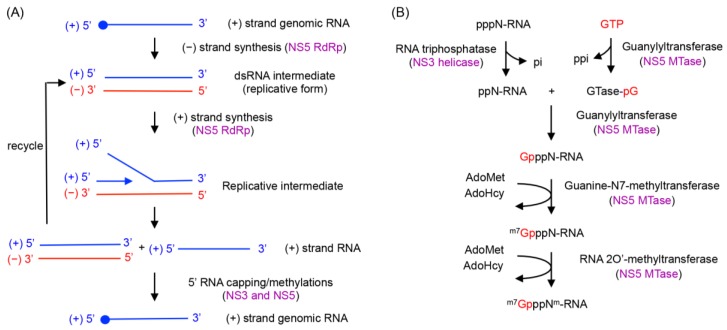
RNA synthesis by the flavivirus replication complex. (**A**) RNA replication by flaviviral NS3 and NS5 proteins. A (+) strand genomic RNA serves as a template to produce (−) strand RNA. The (−) strand RNA exists as a dsRNA intermediate (replicative form). The (−) strand within the dsRNA intermediate is then used as a template for (+) strand RNA synthesis. The dsRNA product is released and recycled for additional (+) strand synthesis. Flavivirus replication is asymmetric, and multiple copies of (+) strand RNA are synthesized from a (−) strand template. The (+) strand RNA is then capped and methylated by NS3 and NS5 to form (+) strand genomic RNA. The identities of the enzymes involved in each step are shown in purple. (**B**) 5′-RNA cap synthesis by flaviviral NS3 and NS5 proteins. The type 1 cap is formed on (+) strand RNA via the four sequential enzyme activities of RNA triphosphatase, guanylyltransferase, guanine-N7-MTase, and RNA 2′O-MTase. First, triphosphatase activity of the NS3 helicase releases the terminal phosphate from the 5′-triphosphate end of (+) strand RNA. A guanosine monophosphate (GMP) moiety from GTP is transferred to the 5′ end of the now-diphosphorylated RNA through guanylyltransferase activity of the NS5 MTase. The capped RNA is then methylated first at the N7 position of the guanine cap and subsequently at the ribose 2′-O position of the first RNA nucleotide. The MTase domain of NS5 carries out both methylations using *S-*adenosyl-l-methionine (AdoMet) as a methyl donor. AdoMet is converted to *S*-adenosyl-l-homosysteine (AdoHcy) during this process.

The flavivirus replication complex carries out RNA synthesis, RNA capping and RNA methylation steps to produce the genome with a type 1 cap structure (^m7^GpppN^m^-RNA) at its 5′ end. RNA synthesis in flavivirus is semi-conservative and asymmetric. The (+) sense RNA, which is the same polarity as the viral genome, is predominantly formed over the (−) sense RNA; single-stranded (ss) RNA found in flavivirus-infected cells is (+) sense RNA, and (−) strand RNA is only detected in the dsRNA form [38]. When DENV or Kunjin virus-infected cells were incubated with a radiolabeled NTP (^3^H-UTP or ^32^P-labeled guanosine-5'-triphosphate (GTP)), three radiolabeled RNA products were identified: a double-stranded replicative form (RF) RNA, single-stranded genome length RNA, and the slowest migrating, replicative intermediate (RI) [22,39,40]. RNase treatment of the RI suggested that the RI likely contains growing nascent RNAs on the RF template, which displace a pre-existing strand of the same polarity (Figure 1A). One to ten nascent RNA strands are synthesized on one RF template at a time [29,39]. Based on these observations, a general scheme of RNA synthesis and the associated required enzymatic activities are outlined in Figure 1A. The genomic (+) sense RNA is first used as a template by NS5 RdRp to synthesize a complementary (−) sense RNA. The (−) strand RNA remains base-paired with the (+) strand RNA, resulting in a dsRNA intermediate. The (−) strand within the dsRNA intermediate then serves as the template to generate (+) sense RNA. NS3 helicase activity may be required to unwind the dsRNA. The nascent (+) strand synthesized on the (−) RNA template displaces a pre-existing (+) strand and is released as a dsRNA product. The newly generated dsRNA is then recycled as a template to generate additional copies of (+) sense RNA.

The (+) strand progeny RNA is subsequently capped at its 5′ end and methylated to form a type 1 cap (Figure 1B). The cap is shown to be present only on genomic RNA, and not on the dsRNA intermediate (RF form) in WNV-infected cells [38]. The RNA-capping process is likely to occur as the (+) strand RNA is synthesized during the initial stages of RNA synthesis (Figure 1), but little is known about how flavivirus coordinates RNA synthesis and 5′ end RNA capping. The RNA capping and methylation processes require three enzymatic activities. First, the 5′-triphosphate end of (+) RNA is converted into a 5′-diphosphate by the RNA triphosphatase activity of the NS3 helicase. Second, a GMP moiety from GTP is transferred to the 5′-diphosphate RNA by a guanylyltransferase (GTase). The NS5 MTase domain has weak guanylyltransferase activity that transfers a GMP cap from GTP to the 5′ end of (+) sense RNA [23]. Finally, the capped RNA is first methylated at the N7 position of guanine and then at the ribose 2′-OH position of the first nucleotide of the RNA. The MTase domain of NS5 functions as both the N7-MTase and the 2′O-MTase, and transfers a methyl group from the cofactor AdoMet to the substrate capped RNA in both reactions [24,41].

## 3. Flaviviral Replication Complex

**Figure 2:**
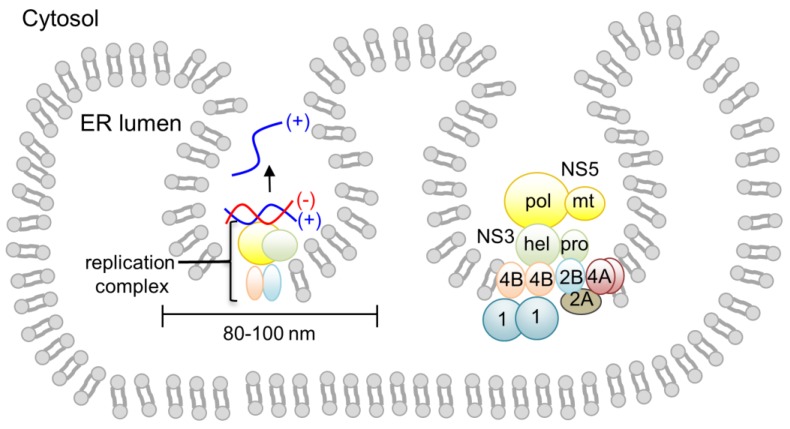
Flaviviral replication complex assembled on the cytoplasmic side of the ER membrane. The viral replication complex is associated with the virus vesicle (single membrane vesicle) formed by invagination of the ER membrane. Interactions between flaviviral NS proteins are indicated schematically. Viral membrane proteins NS2A, NS2B, NS4A, and NS4B form a scaffold for the assembly of NS3 and NS5 proteins. Oligomerization of NS4A and dimerization of NS4B are depicted [51,52]. NS3 interacts with NS2B through its protease domain and with NS4B through its helicase domain. NS5 interacts only with NS3. The NS1 dimer is located in the ER lumen, and associates with NS4A and NS4B.

Genome replication in flavivirus is carried out by a membrane-bound viral replication complex consisting of viral NS proteins, viral RNA and unidentified host proteins [9]. Membrane fractions prepared from the lysates of flavivirus-infected cells contained virus-specific RNA and proteins, and retained all of the RdRp activity [29]. Flaviviruses alter host cellular membrane structures, presumably to protect the viral RNA and replication proteins from triggering the host immune response and being degraded by cytoplasmic enzymes. Using electron microscopy and tomography, flavivirus (DENV, WNV, and TBEV)-infected cells have been shown to form spherical single-membrane vesicles of 80–100 nm in diameter via invagination of the ER membrane into the ER lumen [25,42,43] (Figure 2). This contrasts to the related hepatitis C virus, which shows the double membrane vesicle structures with varied sizes between 150–1000 nm [44,45]. Viral NS proteins NS1, NS2B, NS3, NS4A, NS4B and NS5 along with dsRNA are located in the membrane vesicles, suggesting that these vesicles are the sites of RNA replication [28,45]. The vesicles have a pore connecting the interior of the vesicle to the cytoplasm, which is thought to allow exchange of nucleotides and RNA product with the cytoplasm [25,42]. In addition, virus particles have been shown to bud out on ER membranes next to the replication vesicles, suggesting that the viral replication and encapsidation of the viral genome/virion assembly are likely coordinated.

Although the exact composition of the replication complex is not known, all flaviviral NS proteins have been shown to be a component of the replication complex. Using immunoprecipitation, yeast two-hybrid, and fluorescence resonance energy transfer (FRET) assays, interactions among NS proteins have been identified [27,46,47,48] (Figure 2). Membrane proteins NS2B, NS4A, and NS4B are likely involved in membrane alterations and/or anchoring the viral replication complex to the membrane [26]. NS2B interacts with the three other membrane proteins, NS2A, NS4A, and NS4B [47]. NS4A and NS4B have been proposed to interact with NS1 by genetic studies [49,50]. Oligomerization of NS4A, and dimerizations of NS4B and NS1 have also been reported [51,52]. NS3 itself does not have any membrane-association or transmembrane region. However, the active protease function of NS3 (in the N-terminal domain) requires the cofactor NS2B, thus NS3 localizes to the membrane as an NS3-NS2B complex [15,47]. NS3 has also been shown to interact with NS4B through its C-terminal helicase domain [53]. NS5 also does not have any membrane-associated region and interacts only with NS3. Thus, NS5 likely accumulates to the membrane via NS3-NS5 interactions. Unidentified host proteins are also involved in viral replication. It has been shown that DENV NS5 protein alone was not able to use a viral dsRNA intermediate (RF form) as a template for RNA synthesis. However, the addition of uninfected cell lysate to the NS5 reaction could restore polymerase activity, suggesting that host proteins are also involved in viral replication [40]. Consequently, an *in vitro* flaviviral replication system, which can use the viral genome to synthesize the methylated, capped RNA product, is currently unavailable.

## 4. Inter- and Intramolecular Coordination between NS5 and NS3 during Genome Replication

At the heart of the flaviviral replication complex are NS3 and NS5, which together account for all enzymatic activities required to amplify the RNA genome and to attach a type 1 cap to its 5′ end (Figure 1). Other viral and host proteins in the viral replication complex would provide additional efficiency and specificity for viral replication. NS5 is involved in RNA synthesis, 5′-RNA cap transfer, and cap methylations. The NS3 helicase domain is involved in dsRNA unwinding and removal of the γ-phosphate at the 5′-RNA prior to RNA capping. However, how NS3 and NS5 activities are coordinated during viral replication is not known. It is possible that individual activities of the two proteins are modulated by mutual interaction with each other as well as with other proteins and viral RNA [23,54,55,56]. Interactions between NS3 and NS5 proteins from several flaviviral species have been shown using pull-down assays from infected cell extracts, *in vivo* fluorescent measurements, and yeast two-hybrid studies [46,47,57]. Their respective binding sites map to the C-terminal domain of NS3 (residues 303–618) and the RdRp domain of NS5 (residues 320–368, which also includes a nuclear localization sequence) [58,59,60].

The physical linkage between the MTase and RdRp domains within NS5 suggests that viral genome replication and 5′-RNA capping may be coordinated between the two domains. Several genetic studies show that mutations in the MTase domain impact RNA replication by the RdRp domain, suggesting that the RdRp and MTase interact during viral genome replication [61,62,63,64]. In addition, a full-length DENV type 2 RNA containing an NS5 chimera, in which the MTase from DENV type 4 is fused to the RdRp from DENV type 2, cannot carry out replication although the MTase from DENV type 2 and 4 NS5 share high sequence identity (~70%) [65]. This suggests that sequence-specific interactions between the MTase and RdRp domains are necessary for viral replication. Consequently, there has been great interest in carrying out structural and biochemical studies to define whether and/or how coordination between these activities may occur, and how coordination may be exploited to design novel antiviral agents effective against flavivirus infection [66,67]. The individual MTase and RdRp domains from several flaviviral sources have been structurally characterized, but until recently there were no available structures of full-length flavivirus NS5. The crystal structures of full-length NS5 from Japanese encephalitis virus (JEV) and DENV were recently reported, and provided a first glimpse into how the MTase and RdRp domains interact.

## 5. Full-Length NS5 Structure and Function

**Figure 3:**
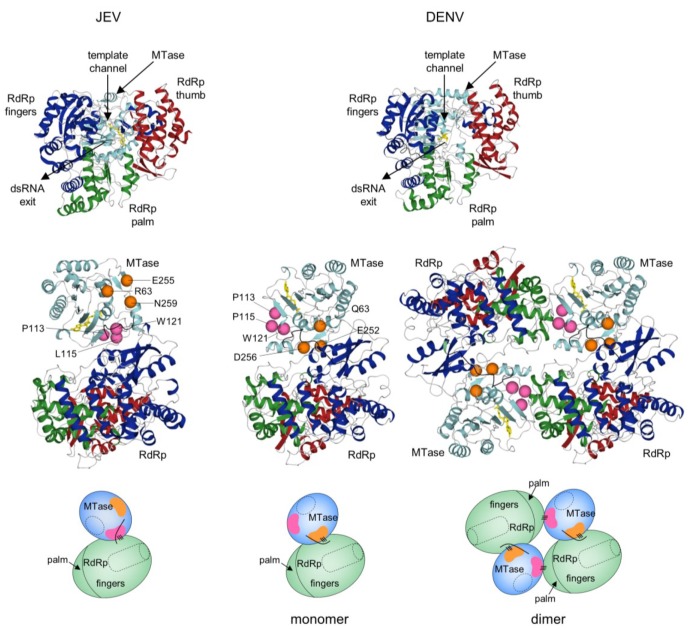
Comparison of full-length NS5 structures from JEV and DENV. Overall fold of JEV and DENV full-length NS5 (Protein Data Bank entries 4K6M and 4VOQ) are shown from the canonical right hand view of RdRp (top) and side view (bottom). Side views of both monomer and dimer forms of DENV NS5 are shown. The RdRp domain consists of fingers (green), palm (blue), and thumb (red) subdomains. The MTase domain (cyan) sits opposite the dsRNA exit channel of the RdRp and near the template-binding channel. The methyl donor used during MTase reaction, AdoMet, is shown in yellow. In each structure, the same region of the RdRp contacts the MTase. The MTase residues at the domain interface from JEV and DENV are indicated by pink and orange spheres, respectively. For comparison, corresponding residues are also mapped on the structures. Domain interactions are also indicated in schematics by a series of three lines below the side view. The MTase active site is indicated by dashed ovals, and the RdRp template channel is shown as a dashed tube.

The recently reported structures of full-length NS5 from JEV and DENV offer details of specific interactions between the MTase and RdRp domains and suggest a structural mechanism by which their activities may be coordinated within the flavivirus replication complex [68,69]. We have recently determined the crystal structure of DENV NS5 as a dimer (in preparation), and will provide an overview of the three structures (Figure 3). Briefly, the MTase domain contains a central core structure characteristic of AdoMet-dependent MTases consisting of a 7-stranded β-sheet surrounded by 4 α-helices. The RdRp domain adopts the shape of a closed right hand and consists of palm, fingers, and thumb subdomains. The inner surfaces of the fingers and thumb subdomains form the entrance to a template-binding channel that leads to the active site in the palm subdomain. The locations of respective active sites in the MTase and the RdRp are indicated in Figure 3. Surprisingly, in both JEV and DENV full-length NS5 the active sites of the MTase and RdRp domains are located on opposite faces and do not interact with each other. The MTase domain sits “behind” the RdRp domain when viewed in its right hand orientation, opposite the dsRNA exit channel and close to the template-binding channel (Figure 3). In all three structures, the individual MTase and RdRp domains are nearly superimposable with one another. The major difference between the JEV and DENV NS5 structures is the relative orientation of the MTase and RdRp domains, primarily due to different conformations of the inter-domain linker (residues 263-272, DENV type 3 numbering). The MTase domains in JEV and DENV NS5 are rotated relative to one another by ~100°. A strictly conserved ^260^Gly-Thr-Arg^262^ (GTR) motif, N-terminal to the inter-domain linker, is proposed to act as a hinge that allows movement of the MTase and RdRp domains relative to one another [68,69,70]. Accordingly, the relative orientation of the MTase and RdRp active sites are quite different in JEV and DENV NS5, and the domain interfaces involve different sets of MTase residues to interact with the same area of the RdRp. In the JEV NS5, the domain interface centers around a hydrophobic core consisting of residues P113, L115, and W121 from the MTase domain, and F467, F351, and P585 from the RdRp. In contrast, the domain interface in DENV NS5 is stabilized mainly by polar and electrostatic interactions involving MTase residues Q63, E252, and D256 to interact with relatively the same area of RdRp (Figure 3). Interestingly, the MTase residues involved in the inter-domain interactions in the JEV NS5 monomer (P113, P115, and W121 in DENV numbering) are present at the dimer interface in our DENV NS5 dimer structure. Thus, the same MTase region mediates intra- and inter-molecular interactions with the RdRp domain in JEV and DENV NS5, respectively, suggesting that NS5 may also function as a dimer (Figure 3). Taken together, comparison of the full-length NS5 structures from JEV and DENV shows that flavivirus NS5 can alter relative MTase and RdRp domain orientations and its monomer-dimer state by modulating its linker region. This may endow NS5 with the flexibility it needs to carry out multiple functions in the replication complex and interact with other proteins at different steps of viral replication (see below).

## 6. Model of Flaviviral Replication

The multifunctional flavivirus NS5 carries out several reactions. These include synthesis of (−) strand RNA, synthesis of (+) strand RNA, addition of a guanine cap to the 5′ end of (+) strand RNA, and two methylation reactions to form a type 1 cap structure at the 5′ end of the nascent RNA. Consequently, NS5 interacts with other viral proteins and several different forms of viral RNA, including both single- and double-stranded, (+) and (−) strand, capped and uncapped, and methylated and unmethylated forms of capped RNA. Thus, NS5 likely needs to adopt multiple conformations during different stages of genome replication and processing, as suggested by the conformational variation observed for the structures of full-length NS5 from JEV and DENV. Below, we summarize viral replication steps in terms of coordination of NS3 and NS5 functions.

**Figure 4:**
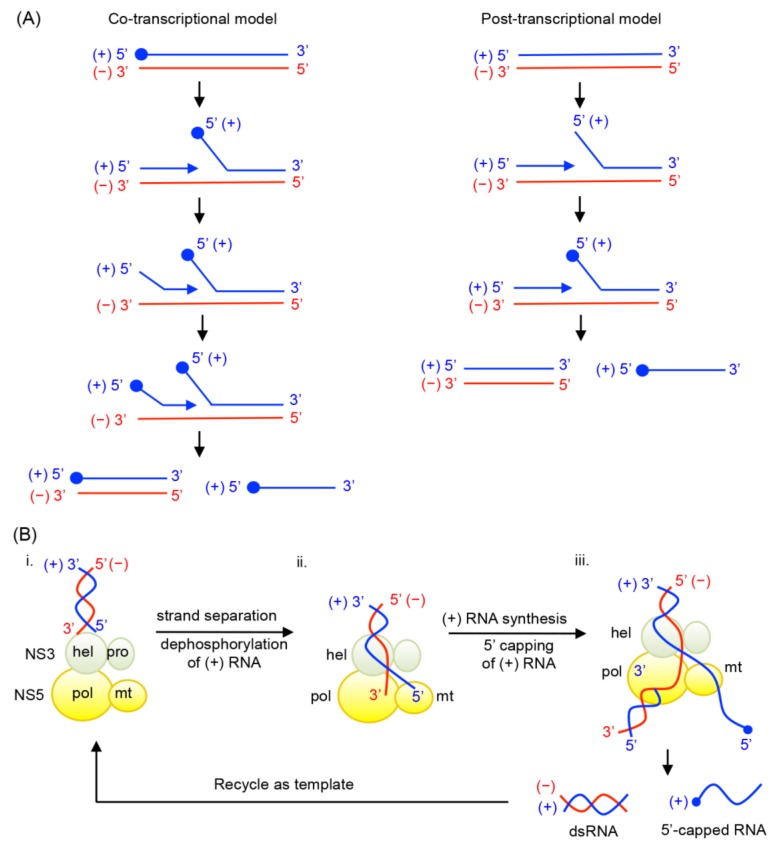
Possible mechanisms of coordination between RNA synthesis and 5′-RNA capping. (**A**) Two possible mechanisms are proposed for 5′-RNA capping for (+) sense RNA. In a co-transcriptional model, 5′-RNA capping occurs co-transcriptionally while nascent (+) sense RNA is being synthesized from dsRNA replicative form. After a short stretch of (+) sense RNA being synthesized, the 5′ end of the nascent (+) RNA would be dephosphorylated by NS3 helicase, and capped and methylated by NS5 MTase. The cap structure is depicted with a closed circle. The 5′-capped (+) sense RNA would then be continuously synthesized until the entire (−) strand is copied. Upon completion of a cycle, a dsRNA containing a capped (+) strand and a capped (+) RNA are synthesized. In a post-transcriptional model, 5′-RNA capping occurs on the fully synthesized (+) strand RNA. Following strand separation of dsRNA, the (+) sense RNA could be dephoshphorylated by NS3 helicase, and capped and methylated by NS5 MTase. Upon completion of a cycle, a dsRNA and a capped (+) RNA are synthesized. (**B**) Coordination of RNA synthesis and 5′-RNA capping by flavivirus NS3 and NS5 in the post-transcriptional model. (**i**) NS3 helicase unwinds the dsRNA intermediate into (+) and (−) sense RNA, and hydrolyzes the 5′ end of (+) sense RNA by its 5′-RNA triphosphatase activity. (**ii**) The 3′ end of the (−) strand RNA will enter the NS5 RdRp template-binding channel, and serve as a template for (+) strand RNA synthesis. The 5′-dephosphorylated (+) RNA enters the NS5 MTase active site for 5′-RNA capping and methylations. (**iii**) Upon completion of nascent (+) sense RNA synthesis, a dsRNA and a 5′-capped (+) strand RNA are released from the NS5 RdRp and MTase domains. The dsRNA product, consisting of the (−) strand template and nascent (+) strand, is then recycled for another round of (+) strand RNA synthesis.

1. (−) strand RNA synthesis: (−) sense RNA synthesis is carried out by NS5 polymerase using the viral (+) RNA genome as a template. The function of NS3 does not seem to be required for (−) strand RNA synthesis, since NS5 (either the full-length protein or the polymerase domain) alone is capable of synthesizing RNA using viral subgenomic RNA. The (+) sense RNA genome contains cyclization sequences at the 5′- and 3′-UTR, and the cyclized genome serves as the template for (−) strand RNA synthesis [1,2,3,4,5,6,7,8,71]. NS5 polymerase recognizes a conserved RNA structure called stem loop A (SLA) within the 5′-UTR as a promoter, and initiates (−) strand RNA synthesis at the 3′ end of the genome. The nascent (−) RNA product exists as dsRNA, base-paired with the (+) strand RNA template [29].
2. (+) strand RNA synthesis: The viral replication complex uses the (−) strand RNA in the dsRNA intermediate as the template to synthesize multiple copies of (+) sense RNA. Both NS5 and NS3 activities are required for (+) RNA synthesis (Figure 4). NS3 helicase first unwinds the dsRNA intermediate into (+) and (−) sense RNAs. Following strand separation, the 3′ end of (−) sense RNA will bind the template-binding channel of NS5 RdRp and serve as a template for (+) strand RNA synthesis. Because (−) strand RNA only exists in the dsRNA form, specific RNA interactions, such as those between the cyclization sequences or stem loop structures necessary for (−) strand synthesis, are not required for (+) sense RNA synthesis [29,72]. Upon completion of nascent (+) sense RNA synthesis, a dsRNA product (consisting of the nascent (+) strand and the (−) strand template) will be released from the RdRp and recycled for another round of (+) strand RNA synthesis [29,36].
3. 5′-RNA capping and methylation of (+) sense RNA: The (+) sense RNA will be capped and methylated to form a type 1 cap at the 5′ end (Figure 1B). NS3 helicase hydrolyzes the 5′-terminal phosphate of (+) sense RNA and converts it to a diphosphate using its RNA triphosphatase activity. The 5′-diphosphorylated (+) sense RNA then binds the NS5 MTase for capping and methylations. For 5′-RNA capping, NS5 MTase first reacts with GTP to form a covalently linked GMP-enzyme intermediate, and then transfers the GMP moiety to the 5′-diphosphate of the (+) strand RNA [23]. Next, the MTase methylates the N7 position of the guanine cap and the ribose 2′-OH position of the first nucleotide. Each methylation reaction requires distinct RNA sequences and lengths, and N7 cap methylation requires the presence of the stem loop A in the 5′-UTR of the viral genome [73]. Since the NS5 MTase carries out three separate reactions using one active site, the 5′ end of (+) RNA needs to dissociate and then re-associate with the MTase at each step [74].
4. Coordination of (+) RNA synthesis and 5′-RNA capping: The 5′-RNA capping and methylation steps are likely coupled with (+) sense RNA synthesis, but during which step of RNA synthesis 5′-RNA capping and methylation occur is not clear. Capping of 5′-RNA could occur on the nascent (+) strand RNA while it is being synthesized by the NS5 RdRp domain (co-transcriptional model in Figure 4A). After a short stretch of (+) sense RNA is synthesized, the 5′ end of the nascent (+) RNA could be dephosphorylated by NS3 helicase, and capped and methylated by NS5 MTase. The 5′-capped (+) sense RNA will then be continuously synthesized until the entire (−) strand is copied. Alternatively, the 5′-RNA capping could occur on the preexisting full-length (+) RNA that is separated from the (−) strand template RNA by NS3 helicase (post-transcriptional model in Figure 4A). Upon unwinding of dsRNA, NS3 helicase hydrolyzes the 5′-terminal phosphate of (+) sense RNA and NS5 MTase subsequently attaches the type 1 cap to the 5′ end. The consequential difference between the two mechanisms would be whether the (+) strand RNA in the dsRNA form is capped. In the co-transcriptional model, all (+) strand RNA, either ssRNA or dsRNA, would be capped and methylated because the nascent RNA is co-transcriptionally capped and methylated. In the post-transcriptional model, only displaced (+) strand RNA, and not the (+) sense RNA in the dsRNA form, would be capped and methylated. Since the cap is shown to be present only on genomic (+) sense RNA, and not on the dsRNA form in WNV-infected cell [29,38], it seems likely that 5′-capping occurs on the fully synthesized (+) sense RNA. In this case, upon completion of a cycle of nascent (+) sense RNA synthesis, a dsRNA product and a capped (+) strand RNA (identical to the viral genome) will be released from the RdRp and MTase domains of NS5 (Figure 4B).

## 7. Conclusions and Future Perspectives

The identification and visualization of subcellular membrane structures used by flavivirus as replication factories have provided insight into how flavivirus may orchestrate viral replication and virion assembly in infected cells. Additionally, crystal structures of the full-length NS5 have provided details of intra- and inter-molecular interactions that stabilize different forms of NS5. These forms may interact with RNA and protein components of the replication complex differently and thus serve as a platform to promote different steps during genome replication. Future studies will be geared toward understanding how these membrane vesicles are related to the assembly and function of the viral replication complex at the molecular level. In particular, how flaviviral and host proteins establish such elaborate membrane vesicles for replication, how flaviviral NS proteins are assembled in the membrane-bound viral replication complex, and how the viral replication complex carries out individual steps of RNA replication are of great interest.
